# Arctiin blocks hydrogen peroxide-induced senescence and cell death though microRNA expression changes in human dermal papilla cells

**DOI:** 10.1186/0717-6287-47-50

**Published:** 2014-09-30

**Authors:** Seunghee Bae, Kyung Mi Lim, Hwa Jun Cha, In-Sook An, Jeong Pyo Lee, Kwang Sik Lee, Ghang Tai Lee, Kun Kook Lee, Ho Jung Jung, Kyu Joong Ahn, Sungkwan An

**Affiliations:** Korea Institute for Skin and Clinical Sciences, Konkuk University, 120 Neungdong-ro, Gwangjin-gu, Seoul, 143-701 Republic of Korea; Coreana Cosmetics Co., Ltd, Cheonan-si, Chungcheongnam-do 330-833 Republic of Korea; Department of Dermatology, Konkuk University School of Medicine, 120 Neungdong-ro, Gwangjin-gu, Seoul, 143-701 Republic of Korea

**Keywords:** Dermal papilla cell, Senescence, Cell death, microRNA, Arctiin

## Abstract

**Background:**

Accumulating evidence indicates that reactive oxygen species (ROS) are an important etiological factor for the induction of dermal papilla cell senescence and hair loss, which is also known alopecia. Arctiin is an active lignin isolated from *Arctium lappa* and has anti-inflammation, anti-microbial, and anti-carcinogenic effects. In the present study, we found that arctiin exerts anti-oxidative effects on human hair dermal papilla cells (HHDPCs).

**Results:**

To better understand the mechanism, we analyzed the level of hydrogen peroxide (H_2_O_2_)-induced cytotoxicity, cell death, ROS production and senescence after arctiin pretreatment of HHDPCs. The results showed that arctiin pretreatment significantly inhibited the H_2_O_2_-induced reduction in cell viability. Moreover, H_2_O_2_-induced sub-G1 phase accumulation and G2 cell cycle arrest were also downregulated by arctiin pretreatment. Interestingly, the increase in intracellular ROS mediated by H_2_O_2_ was drastically decreased in HHDPCs cultured in the presence of arctiin. This effect was confirmed by senescence associated-beta galactosidase (SA-β-gal) assay results; we found that arctiin pretreatment impaired H_2_O_2_-induced senescence in HHDPCs. Using microRNA (miRNA) microarray and bioinformatic analysis, we showed that this anti-oxidative effect of arctiin in HHDPCs was related with mitogen-activated protein kinase (MAPK) and Wnt signaling pathways.

**Conclusions:**

Taken together, our data suggest that arctiin has a protective effect on ROS-induced cell dysfunction in HHDPCs and may therefore be useful for alopecia prevention and treatment strategies.

## Background

Reactive oxygen species (ROS) are reactive oxygen-containing endogenous byproducts that are produced during normal metabolism and play pivotal roles in maintaining homeostasis [1]. Under stressful environmental conditions (e.g., ionizing radiation, ultraviolet (UV) radiation, drugs, and smoke), the levels of ROS, including hydrogen peroxide (H_2_O_2_), are significantly increased [2]. These ROS subsequently activate various signaling molecules, including p53 and mitogen-activated protein kinase (MAPK) to induce cell growth arrest and apoptosis [3, 4]. In skin, exogenous ROS can cause cellular damage, impaired collagen synthesis, and keratinocyte apoptosis, which are mainly observed in aged skin [5]. One group reported that high ROS levels are involved in the etiologies underlying vitiligo skin disease and skin cancer [6]. Furthermore, accumulating evidence supports the hypothesis that oxidative stress caused by H_2_O_2_ is a key factor in the onset and progression of hair loss, which is known as alopecia [7–9].

Alopecia is a hair loss skin disorder that typically causes baldness [10]. It was originally defined as an age-dependent or androgen-dependent mechanism in dermal papilla cells (DPCs) [11]. However, clinical reports have demonstrated that alopecia does not occur in an androgen-dependent manner; there are also androgen-independent mechanisms, including chemotherapy-dependent and stress-dependent effects [9, 12, 13]. A growing number of studies have shown that the levels of apoptosis and senescence are significantly increased in balding DPCs as compared with non-balding DPCs [7, 14]. Interestingly, ROS has been known as an important inducer of the androgen-dependent and -independent alopecia [9]. The dermal papillae of balding scalps showed higher levels of ROS compared with non-balding scalp [15, 16]. Moreover, increased ROS levels were associated with decreased DPC motility [7]. In addition, DPCs from balding scalp also exhibited higher levels of cell senescence [7]. More recent studies also demonstrated that cisplatin-induced alopecia is mediated by ROS production and ROS-mediated apoptosis in DPCs [17]. These finding have led to the hypothesis that ROS may be an important target when designing therapeutic strategies to prevent or treat alopecia.

Arctiin is a lignin chemical reagent isolated from *Arctium lappa*. This chemical reagent has been known to exert anti-inflammatory, anti-proliferative, and anti-microbial effects [18–21]. We recently reported that arctiin has a protective effect against UVB radiation in skin cells, including dermal fibroblasts and keratinocytes [22, 23]. We subsequently observed that the arctiin-mediated anti-photoaging effect is functionally related with microRNA (miRNA)-mediated signaling pathways [22, 23]. However, it is unknown whether arctiin exerts anti-oxidative effects and what biological effects arctiin has on human follicle cells. In present study, we studied the biological events of human hair dermal papilla cells (HHDPCs) in the presence of arctiin and/or H_2_O_2_ and performed cell-based assays to determine whether H_2_O_2_-induced cell dysfunction could be inhibited by arctiin treatment. We also investigated the role of miRNA-mediated mechanisms in using microarrays and bioinformatic analysis.

## Results

### Arctiin inhibits H_2_O_2_-mediated cell proliferation loss in a dose-dependent manner in HHDPCs

**Figure 1:**
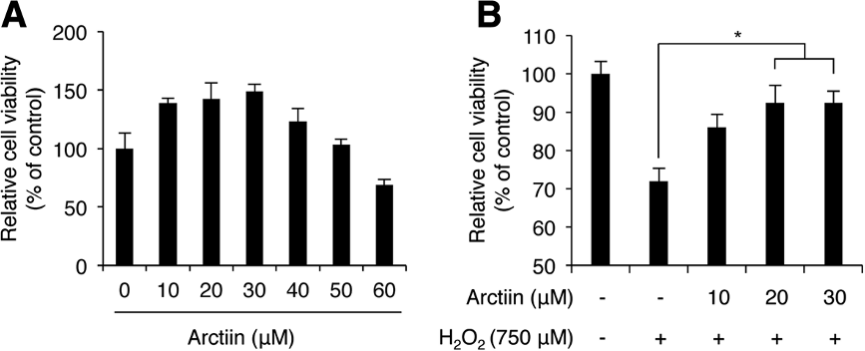
**H**_**2**_ **O**_**2**_ **-mediated loss of HDPPC viability was rescued by arctiin treatment. (A)** WST-1 cytotoxicity assays of HHDPCs treated with various doses of arctiin. Each bar represents the mean ± SD from three independent experiments. **(B)** Arctiin dose-dependently inhibited H_2_O_2_-induced cytotoxicity as measured with WST-1 assays. The graph represents the mean ± S.D. of relative cell viability from triplicate experiments. *p < 0.05 compared with H_2_O_2_-treated HHDPCs.

Before we analyzed the inhibitory effect of arctiin against H_2_O_2_-induced cell dysfunction, we preferentially sought to evaluate the effect of arctiin on HHDPC growth. First, HHDPCs were exposed to different concentrations of arctiin for 24 h, and cell viability was analyzed with WST-1 assays. As shown in Figure 1A, 10, 20, and 30 μM arctiin significantly increased cell viability by 38.89 ± 3.99%, 42.48 ± 13.69%, and 48.96 ± 6.07% compared with dimethyl sulfoxide (DMSO)-treated control cells. However, exposure to larger doses of arctiin (≥40 μM) did not increase cell viability beyond that observed in cells treated with 10 μM arctiin, and 60 μM arctiin induced HHDPC cytotoxicity. Therefore, we concluded that an arctiin dose of 10 to 30 μM arctiin has maximum growth-promoting effect on HHDPCs, and those doses were used in further experiments.

Next, we investigated the inhibitory effect of arctiin on H_2_O_2_-induced cell dysfunction. We first performed WST-1 assays to analyze the inhibitory effect of H_2_O_2_ on HHDPC viability. Cells were pretreated with 0 to 30 μM arctiin for 8 h and then stimulated with 750 μM H_2_O_2_. Cell viability was analyzed after 24 h. As expected, arctiin pretreatment attenuated the H_2_O_2_-induced decrease in cell viability in a dose-dependent manner (Figure 1B). DMSO and H_2_O_2_-treated cells showed a 38.15 ± 3.52% reduction in cell viability compared with untreated cells; however, 10, 20, and 30 μM arctiin-pretreated and H_2_O_2_-posttreated cells showed only 13.95 ± 3.34%, 7.49 ± 4.40%, and 7.54 ± 2.68% reductions compared with untreated cells, respectively. These results indicate that arctiin attenuates H_2_O_2_-mediated cytotoxicity in HHDPCs.

### Arctiin inhibits H_2_O_2_-mediated cell death and cell cycle arrest in HHDPCs

**Figure 2:**
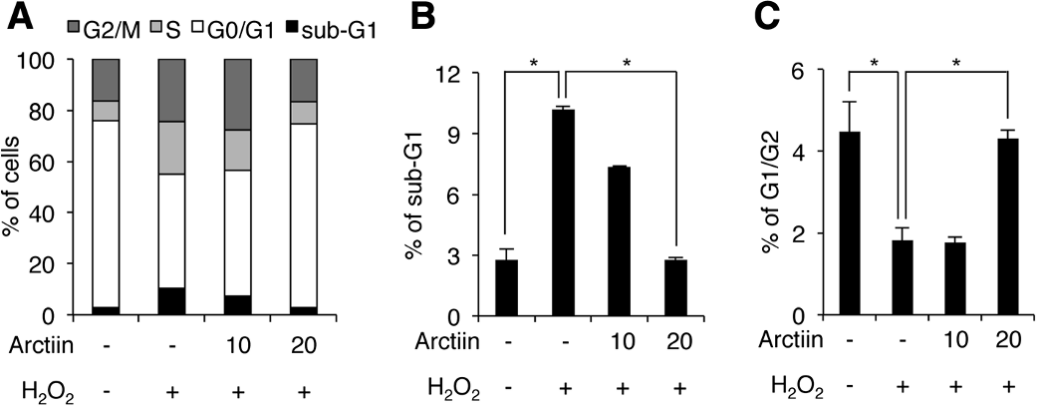
**H**_**2**_ **O**_**2**_ **-induced G2 arrest and cell death were rescued by arctiin. (A)** HHDPCs were treated with DMSO or arctiin prior to H_2_O_2_ exposure, and cell cycle status was assessed flow cytometry. **(B and C)** The graphs represent the mean values of cell populations from three independent experiments (sub-G1 and G1/G2, respectively). *p < 0.05 compared with control or H_2_O_2_-treated HHDPCs.

To confirm the result shown in Figure 1B, we performed cell cycle analyses using PI staining and flow cytometry. HHDPCs were treated with arctiin and H_2_O_2_ under the same conditions used for Figure 1B, and then cells were stained with PI solution to analyze cell cycle patterns. As shown in Figure 2A, arctiin and H_2_O_2_ treatment altered the cell cycle distribution of HHDPCs. Notably, H_2_O_2_ only treatment led to accumulation of 7.45% in sub-G1, 12.84% in S phase and 7.45% in G2/M phase, with a corresponding decrease in the percentage of G0/G1 phase cells as compared with untreated control cells (Figure 2A). We also found that those accumulations in sub-G1, S, and G2/M phases in H_2_O_2_-treated cells were significantly decreased by arctiin pretreatment in a dose-dependent manner. Pretreatment with 10 and 20 μM arctiin led to reductions of 2.82% and 7.43% in sub-G1 phase cells as compared with H_2_O_2_-treated cells, respectively, indicating that arctiin inhibited H_2_O_2_-mediated sub-G1 accumulation, which is indicative of dead cells (Figure 2B). Also, the proportion of G2/G1 cells was increased by H_2_O_2_ treatment as compared with untreated control cells, indicating that H_2_O_2_ induced G2 arrest in HHDPCs (Figure 2C). However, the proportion of G2 arrest cells was significantly decreased by arctiin pretreatment (Figure 2C). Overall, these results suggest that arctiin blocks H_2_O_2_-mediated cell death and G2 arrest in HHDPCs.

### Arctiin inhibits H_2_O_2_-mediated ROS generation in HHDPCs

**Figure 3:**
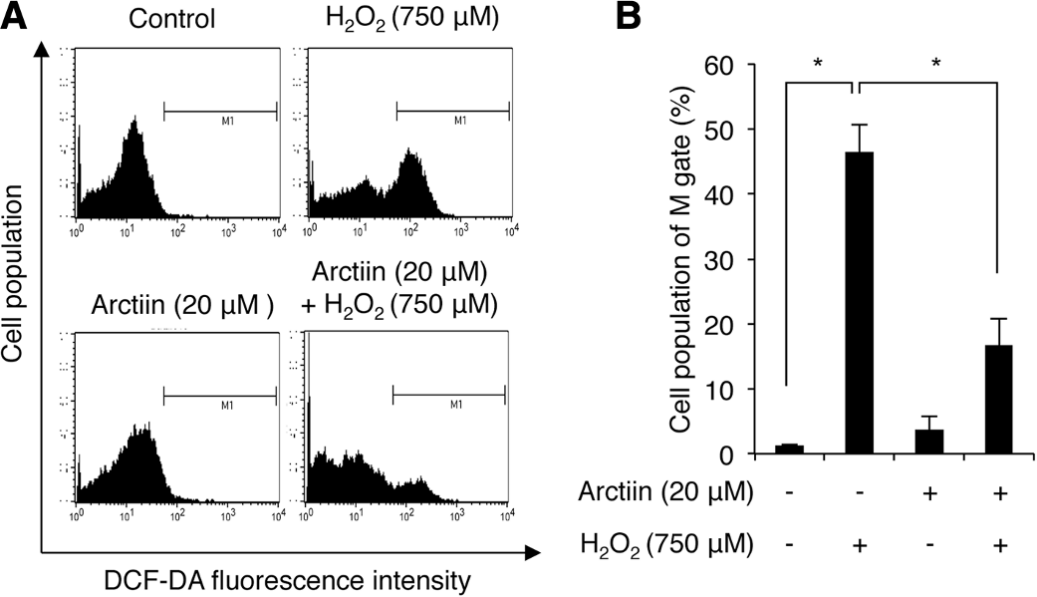
**Analysis of intracellular ROS levels with DCF-DA assays. (A)** HHDPCs were pre-treated with DMSO or arctiin (20 μM) followed by H_2_O_2_ (750 μM) prior to flow cytometry. **(B)** The graph indicates the mean value of M1-phase (DCF-DA-stained) cells from three independent experiments. *p < 0.05 compared with H_2_O_2_-treated HHDPCs.

ROS generation mediated by H_2_O_2_ is characterized by increases in cell death and cell cycle arrest in several cell lines [1]. To determine whether arctiin pretreatment inhibits H_2_O_2_-mediated ROS generation, we performed DCF-DA analyses to assess intracellular ROS production in HHDPCs. As shown in Figure 3A, arctiin did not alter intracellular ROS levels in untreated control cells, but it significantly abolished the H_2_O_2_-induced increase in intracellular ROS generation. Cells treated with 750 μM H_2_O_2_ showed a 45.77% accumulation of M phase (DCF-positive) cells as compared with untreated control cells (Figure 3B). However, pretreatment with 20 μM arctiin, led to reduction of 29.77% of cells in the M phase as compared with H_2_O_2_-treated cells (Figure 3B). These results suggest that H_2_O_2_-mediated ROS production in HHDPCs is inhibited by arctiin.

### Arctiin inhibits H_2_O_2_-mediated senescence in HHDPCs

**Figure 4:**
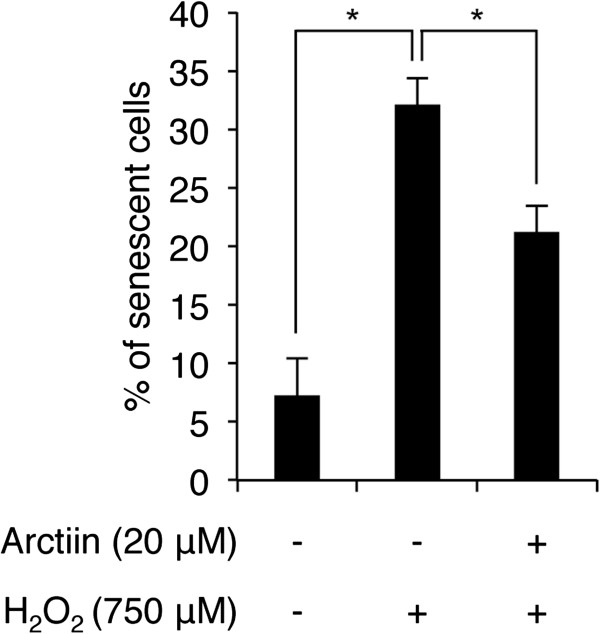
**Effect of arctiin on H**_**2**_ **O**_**2**_ **-induced senescence as measured by SA-β-gal assays.** HHDPCs were pre-treated with DMSO or arctiin (20 μM) followed by H_2_O_2_. The graph indicates the mean value of senescent (SA-β-gal stained) cells from three independent experiments. *p < 0.05 compared with H_2_O_2_-treated HHDPCs.

Cell cycle arrest in the G2 phase and ROS generation are functionally related with cellular senescence [24, 25]. Therefore, we next determined whether G2 cell cycle arrest and ROS generation contribute to senescence and whether arctiin pretreatment can block H_2_O_2_-mediated senescence in HHDPCs. Using SA-β-galactosidase (SA-β-gal) assays, we evaluated cellular senescence by counting the SA-β-gal-positive blue-stained senescent cells after arctiin and/or H_2_O_2_ treatment under the same experimental conditions shown in Figure 3A. H_2_O_2_ treatment increased the percentage of senescent cells by 24.89% compared with untreated control cells, however, 20 μM arctiin pretreatment the percentage of senescent cells by 10.89% compared with H_2_O_2_-treated cells (Figure 4). These results indicate that arctiin negatively regulates H_2_O_2_-mediated senescence in HHDPCs.

### Arctiin alters H_2_O_2_-mediated changes in miRNA expression

miRNAs are important small non-coding RNA molecules, and exerts their biological functions by posttranscriptionally regulating those of their target genes [26, 27]. Numerous studies have demonstrated that miRNAs regulate 4 major biological functions, such as development, proliferation, differentiation and apoptosis [28, 29]. Furthermore, accumulating results have shown that altered miRNA expression profiles are involved in UVB- or H_2_O_2_- protective effects and even androgenetic alopecia in human skin cells [23, 30–32]. Therefore, we next sought to understand the putative molecular mechanism underlying the protective effect of arctiin against H_2_O_2_ in HHDPCs by analyzing miRNA expression changes. We performed miRNA microarrays and identified 30 miRNAs that were differentially expressed following arctiin pretreatment and H_2_O_2_ exposure as compared with H_2_O_2_-treated cells. Eighteen and 12 miRNAs were upregulated and downregulated more than 2.0-fold, respectively (Table 1). The most dysregulated miRNAs were miR-602 (5.74-fold increase) and miR-1290 (5.80-fold decrease). These findings indicate that arctiin regulates the expression levels of specific miRNAs in HHDPCs.

**Table 1:** **Significantly altered miRNAs (>2-fold change) following arctiin treatment of H**_**2**_ **O**_**2**_ **-exposed HHDPCs**

miRNA	Change relative to controls	Direction of regulation	Chromosome	miRNA	Change relative to controls	Direction of regulation	Chromosome
hsa-miR-1181	2.13	Up	19	hsa-miR-874	2.97	Up	5
hsa-miR-125a-5p	5.04	Up	19	hsa-miR-890	2.83	Up	X
hsa-miR-21-3p	2.82	Up	17	hsa-miR-939	2.59	Up	8
hsa-miR-29b-1-5p	3.12	Up	7	hsa-miR-1290	−7.56	Down	1
hsa-miR-3663-3p	2.19	Up	10	hsa-miR-1915-3p	−2.63	Down	10
hsa-miR-3127-5p	2.01	Up	2	hsa-miR-2861	−3.31	Down	9
hsa-miR-3663-3p	2.03	Up	10	hsa-miR-3665	−2.37	Down	13
hsa-miR-371a-5p	3.14	Up	19	hsa-miR-4257	−3.62	Down	1
hsa-miR-4327	2.95	Up	21	hsa-miR-452-5p	−2.54	Down	X
hsa-miR-584-5p	2.31	Up	5	hsa-miR-513a-5p	−3.15	Down	X
hsa-miR-602	5.74	Up	9	hsa-miR-572	−5.80	Down	4
hsa-miR-629-3p	2.71	Up	15	hsa-miR-629-3p	−3.03	Down	15
hsa-miR-642b-3p	2.10	Up	19	hsa-miR-765	−7.18	Down	1
hsa-miR-651	3.91	Up	X	hsa-miR-875-5p	−3.91	Down	8
hsa-miR-762	2.84	Up	16	hsa-miR-940	−2.31	Down	16

To investigate the cellular effect of the altered miRNAs on HHDPCs, we selected the altered specific miRNAs and gathered the list of putative target mRNAs of the miRNAs using a target prediction tool (MicroCosm Targets ver. 5), and then determined the biological functions associated with the target genes by Gene Ontology (GO) analysis using AmiGO bioinformatic tools. Our data show that arctiin has a protective effect against H_2_O_2_-induced cellular senescence and apoptosis in dermal papilla cells; therefore, we analyze GO of the target genes and categorize them into cellular processes including aging, skin development, apoptosis and cell proliferation. As shown in Tables 2 and 3, the altered miRNAs were functionally related in the four biological categories. Mir-602 was the most upregulated miRNA and has many targets including EDN1 and SOD2 (aging); APC (skin development); ERBB4, PPARG, and TP53BP2 (apoptosis); and STAT3, CDK9, and ID4 (cell proliferation). Mir-1290 was the most downregulated miRNA and targets SLC1A2 (aging), APC and COL8A1 (skin development), NOTCH1 and BMI1 (apoptosis), and ROBO1, CDC27 (cell proliferation).

**Table 2:** **Predicted targets of miRNAs upregulated by arctiin in H**_**2**_ **O**_**2**_ **-treated HHDPCs**

miRNA	Target genes and functions			
	Aging	Skin development	Apoptosis	Cell proliferation
hsa-miR-1181	-	-	-	-
hsa-miR-125a-5p	EPO, EDN1, BCL2, BAK1, CASP2, PTH1R	COL4A3	ITCH, COL4A3, HIPK2, RAF1, EPO, EDN1, BCL2, MAP2K7, MAP3K1, ARHGEF3, TRAF6, CASP2, BAK1, IRF1, APPL1, ETS1, BMF, MCL1, BCL2L2, BAG4, HK2, FOXQ1, CREB1, E2F2, TP73, USP7, MAPK7, FGFR2, BRCA1, MAP3K11, REST, DICER1, ARHGEF2, MAP3K10, SORT1, BNIP2, DDX5, CSNK2A1, MKNK2	BRCA1, MAP3K11, ETS1, LIF, SOX11, CD34, NCOR2, BAP1, IRF1, APPL1, REST, DICER1, EPO, EDN1, BCL2, BAK1, ARHGEF2, FGFR2, ITCH, ABCB1, COL4A3, KIF15, HIPK2, RAF1, EIF5A2, CARM1
hsa-miR-21-3p	CDK6	-	MAP2K4, MAP3K1, BCL2L11, ROBO2, RNF41, BAG4, CUL3, FOXO3, SMAD3, AMIGO2, KDM28, DAB2IP, TRIM32, SOX4, CCAR1, SLC11A2, DSG1	CDK6, KDM28, CUL3, SOX4, SMAD3, NR6A1, FTO, CD274, DAB2IP, TRIM32, FOXO3, PBRM1
hsa-miR-29b-1-5p	NR3C1, SIRT1	-	NR3C1, SIRT1, REST, PTK2, SOS2, NUAK2, PSMD7	NR3C1, SIRT1, REST, PTK2, FGF18, INSR, PBRM1
hsa-miR-3663-3p	FAS, CASP2, CDKN1A, PTH1R	ADAMTS2, BCL11B, COL3A1, COL1A1	CDKN1A, BCL11B, PPP2R1B, TGFB2, DDX5, COMP, PIGT, FAS, USP28, TIAL1, CASP2, PSMA2, MEF2D	FAS, USP28, TIAL1, TGFB2, CDKN1A, BCL11B, VSIG
hsa-miR-371a-5p	-	LEF1, ATP7A, COL8A1	LEF1, SOX2, CITED2, STK4, RB1CC1, BARD1, RPS6KA1, GSK3B, PSMF1, MAP3K1, NR4A2, DYRK2, ITSN1,	LEF1, SOX2, CITED2, STK4, COL8A1, RNF10, MAPRE1, BTG3, CCR2, FRS2, PRMT5
hsa-miR-4327	RPS6KB1	STS	RPS6KB1, ADAMTS20, FGF10, IGF1R, FGD4, RPS6KA3	RPS6KB1, IGF1R, FGF10, STS, PROX1, NF2, IFNK
hsa-miR-584-5p	SOD2, MORC3	PTCH2	SOD2, SIX1, LRP6, FGF10, TRIM24, HDAC1, CUL2, SRPK2, NBN, ETS1, DSG3, MTDH, PDE3A, SLAMF7	SOD2, SIX1, LRP6, FGF10, TRIM24, HDAC1, CUL2, SRPK2, NBN, ETS1, PRRX1, FER, USP8, WWTR1
hsa-miR-602	EDN1, VDR, SOD2, HTT, SLC34A2, CHEK1	APC	EDN1, VDR, SOD2, HTT, APC, NOG, ERBB4, PIM1, PPARG, ALDH1A2, CLI2, SEMA3A, H1F0, DYRK2, BCL2L15, JMY, PSMD2, TP53BP2, MYO18A, SHF	NOG, ERBB4, PIM1, PPARG, CLI2, CDK13, LIFR, STAT3, EDN1, VDR, SOD2, CDC27, EMX2, CDK9, RTKN2, ID4, APC, ALDH1A2, PPP1R8, ACSL6, ZEB1
hsa-miR-642b-3p	HMGA2, PTEN, CDKN1A, SERPINE1, MET	BCL11B	SERPINE1, BCL11B, PDCD10, HMGA2, PTEN, RB1, CYLD, EIF2AK2, BCLAF1, MAPK8, CDKN1A, WT1, CDK5R1, MTCH1, EPHA7, NR4A3, CSNK2A1	SERPINE1, BCL11B, PDCD10, HMGA2, PTEN, WT1, AMBN, CDKN1A, CDK5R1, RB1, PAX6, NR2F2, PHOX2B
hsa-miR-651	-	-	BTC, MED1, ATG3, CHMP3	CTC, MED1
hsa-miR-762	RELA, PML	-	RELA, PML, MAPK1, SOX7, ITCH, HIPK2, BCL6, PPARD, CX3CL1, AGAP2, MYO18A, PAK4, ABR, CLIP3, ADD1, PAX7, ITGB2, PDE1B, MAP3K9	EFNB1, LIFR, MMP14, BAP1, RELA, PML, MAPK1, SOX7, PAK4, ITCH, HIPK2, BCL6, CX3CL1, AGAP2, PPARD, NRARP, PTCH1, WARS, FTO
hsa-miR-874	DDC	-	ESR1, ALDH1A2, HIPK2, PAK7, GZMB, IKBKB, SORT1	ESR1, ALDH1A2, HIPK2, PAK7, RXRB, COMT
hsa-miR-890	CDK6, SERP1, F3, PDCD4, ATP5G3, TIMP3	TCF7L2, ITGA2, ERRFI1	SORT1, UBE2B, MAX, PEG3, F3, PDCD4, TCF7L2, SNAI2, AIPL1, AKAP13, ALB, TRIO, REST, PROK2, KRIT, NF1, APBB1, PSME2	F3, CDK6, TCF7L2, SNAI2, REST, PROK2, KRIT, NF1, EGF, SOX17, MARCKSL1, WARS, EPS8,
hsa-miR-939	TIMP1, ATM, CDKN1A, NEK6, SCL34A2, PRELP, SLC1A2	NGFR, COL1A1	TNF, BCL6, BTC, NRG1, IHH, THRA, IP6K2, PAX7, CASP10, CDKN1A, CAMK1D, TRAIP, WNK3, CLIP3, MT3, INHBB, TIMP1, ATM, NEK6, NGFR, NACC1, USP7, CSNK2A2, BCL2L2	BCL6, BTC, NRG1, IHH, GRN, TRAIP, CDKN1A, TNF, E2F8, RXRB, RARA, DRD2, CSF1, TIMP1, ATM, NGFR, MT3, NOS2, AGGF1, ELN

**Table 3:** **Predicted targets of miRNAs downregulated by arctiin in H**_**2**_ **O**_**2**_ **-treated HHDPCs**

miRNA	Target genes and functions			
	Aging	Skin development	Apoptosis	Cell proliferation
hsa-miR-1290	HMGA2, NUAK1, TERF2, SLC1A2, FADS1, DDC	APC, COL8A1	HMGA2, APC, RRN3, ITGAV, CSE1L, NOTCH1, GAS, BMI1, FOXC1, ROBO1, USP28	HMGA2, BMI1, NUAK1, APC, MLL2, RRN3, ITGAV, CSE1L, NOTCH1, GAS, FOXC1, ROBO1, USP28, CDC27, HES1, COL8A1, NPR3, CDKN2B, FIGF, NRAS
hsa-miR-1915-3p	BCL2, SRF, SREBF2, HSD17B10	SRF, DDR1, EDA	BCL2, MAPK311, ARHGEF2, CDK5R1, WNT3A, MMP9, THRA, MEF2D, SET, CD44	DDR1, CDK2, DEAF1, TIRAP, BCL2, MAP3K11, ARHGEF2, CDK5R1, WNT3A, TNFSF11, WDR6, AZGP1, TAL1, DPT
hsa-miR-2861	P2PY2, FADS1	NGFR, EDA	NGFR, PTPRC, ITGA1, MAEL, FGD2, AKT2, PAX8	NGFR, PTPRC, SLAMF1, HOXB4, AKT2, HOXB4,
hsa-miR-3665	AQP2	-	AQP2, FGFR1, BOK, TRIO, NOTCH1, PGAP2, PDPK1, BARD1	BOK, FEZF1, ABCB1, FOXO4, EIF5A2, WNT9A, NDFIP1, FGFR1, NOTCH1, CDK13
hsa-miR-4257	TWIST1, CTSC, HMGA1	COL2A1	TWIST1, CTSC, IGF1R, IL10, GSK3B, RASSF5, COL2A1, AKT1S1, CD44, ASAH2	TWIST1, HMGA1, IL10, INSR, MAPRE2, PA2G4, PRDM1, IGF1R, MMP14, EMX2, TRIM27, MCM7
hsa-miR-452-5p	TIMP3	-	SPRY2, PAX3, SOX7, LRP6, SNAI2, CSNK2A2, FGD4, PKN2, ITGA6, PDCD6IP	SPRY2, PAX3, SOX7, LRP6, SNAI2, RPA1, EPS8, NFIB, MAPRE1, ODZ1, CDCA7L, CD47, E2F3, PURA, RUNX1
hsa-miR-513a-5p	SERP1, NEK6, CDK6, DLD, PRKCD, MAP2K1, MORC3, LRRK2, SLC1A2	WNT7A, SFN, TFAP2B, APC	SPRY2, MLL, GATA3, BCL6, PRKCD, NEK6, MED1, PLK2, WNT7A, SFN, APC, HOXA5, AKAP13, USP47, MAP2K4, ISL1, MITF, STAT1, XIAP, TFAP2B, HDAC2, TRIM2, CREB1, MAP3K7, WNK3, SOS1, FGD4	TFAP2B, WNT7A, APC, HGF, EBXW7, TSC1, KRAS, RXRB, XIAP, SPRY2, GATA3, BCL6, HOXA5, ISL1, MITF, STAT1, NFIB, E2F7, MAGI2, PDKK, HDAC2, MED1, EHF, LIFR, PURA, GATA2, ATF3, VIP, SOX11, RUNX1, RNF139, PCM1, TSHR, EPS8
hsa-miR-572	-	-	-	-
hsa-miR-629-3p	SOD2, VDR, EDN1, CHEK1, SLC34A2	-	THOC1, MYO18A, TP53BP2, SOD2, VDR, EDN1, APC, PPARG, PIM1, ERBB4, PSMD2, PERP, BCL2L15	DLG3, RTKN2, CDK9, STAT3, SOD2, VDR, EDN1, PPARG, PIM1, ERBB4, APC, STAT6, PDGFC, ZEB1, ID4, LIFR, NOLC1, EPHB1, ACSL6, EREG, CDK13, CDC27
hsa-miR-765	VDR, RELA, SOCS3, TIMP3, LOXL2	PDGFA, ERRFI1	DLG5, RARG, MED1, EGLN2, VDR, RELA, SOCS3, ERBB4, RNF41, ATG7, ARHGEF11, PDE5A, CXCL12, GLI3, DIDO1, CASP9, ACIN1	CXCL12, GLI3, DLG5, RARG, VDR, RELA, ERBB4, PDE5A, MED1, PDGFA, CD34, GPC3, PDX1, COL8A1, PES1, TSC2, SF1, FTO, GABBR1,
hsa-miR-875-5p	TGFB3, SMC5	TCF7L2	TGFB3, TCF7L2, EYA1, MDM4, MEF2C, EGFR, WNK3	MDM4, MEF2C, EGFR, FRS2, TGFB3, EYA1, TOB2, TBX18, CEP120, TCF7L2, TIRAP, RNF139, SALL1
hsa-miR-940	-	-	-	-

Previous studies have indicated that arctiin-mediated alterations in miRNAs may be involved in regulated the four cellular mechanisms mentioned above. Also, as a matter of fact, the GO term contains bi-directional processes for each term. The term of ‘apoptosis’ includes both anti-apoptotic and pro-apoptotic processes. Therefore, GO analysis is not sufficient for understanding the biological functions of altered miRNAs in our study. To improve the accuracy of the biological meaning of the target genes, the targets were analyzed using the Kyoto Encyclopedia of Genes and Genomes (KEGG) pathway-based enrichment analysis program in the bioinformatic database DAVID. As shown in Tables 4 and 5, the altered miRNAs may be involved in regulating pathways involved in cancer, cell cycle, and Wnt and MAPK signaling, among others. For example, miR-602 is putatively involved in regulating MAPK and insulin signaling pathways; however, miR-1290 is involved in cancer, focal adhesion, and insulin signaling pathways. Overall, the results indicate that the protective effect of arctiin against H_2_O_2_-induced alterations in HHDPCs may be regulated by arctiin-specific miRNAs and pathways that are possibly affected by miRNAs.

**Table 4:** **Main functions of upregulated miRNAs predicted by bioinformatic analysis**

miRNA	Putative target genes	KEGG pathway	Genes involved in the term	% of involved genes/total genes	***p***-value
(***Homo sapiens***)					
miR-1181	2	-	-	-	-
miR-125a-5p	162	Pathways in cancer	8	4.9	3.60E-02
		Cell cycle	4	2.5	1.20E-01
miR-21-3p	210	Cell adhesion molecules	7	3.3	4.70E-03
		Ubiquitin mediated proteolysis	6	2.9	2.30E-02
		Oocyte meiosis	5	2.4	4.20E-02
miR-29b-1-5p	265	Insulin signaling pathway	5	1.9	8.50E-02
		Cell cycle	4	1.5	2.00E-01
		Wnt signaling pathway	4	1.5	2.90E-01
		Jak-STAT signaling pathway	4	1.5	3.00E-01
miR-3663-3p	305	MAPK signaling pathway	12	3.9	5.90E-03
		Pathways in cancer	11	3.6	5.50E-02
		Focal adhesion	7	2.3	1.30E-01
		Cytokine-cytokine receptor interaction	7	2.3	3.00E-01
miR-371a-5p	351	Spliceosome	8	2.3	4.20E-03
		Wnt signaling pathway	7	2	3.60E-02
miR-4327	112	MAPK signaling pathway	4	3.6	1.20E-01
		Pathways in cancer	4	3.6	1.80E-01
		Melanoma	3	2.7	4.00E-02
		Calcium signaling pathway	3	2.7	1.90E-01
miR-584-5p	288	MAPK signaling pathway	8	2.8	9.70E-02
		Pathways in cancer	8	2.8	2.10E-01
miR-602	302	MAPK signaling pathway	7	2.3	2.20E-01
		Insulin signaling pathway	6	2	5.30E-02
		Alzheimer’s disease	6	2	1.00E-01
		Calcium signaling pathway	6	2	1.30E-01
miR-629-3p	445	Pathways in cancer	10	2.3	2.10E-01
miR-642b-3p	262	Glioma	4	1.5	3.20E-02
		Melanoma	4	1.5	4.30E-02
		Cell adhesion molecules	5	1.8	5.70E-02
miR-651	60	Calcium signaling pathway	3	4.3	5.40E-01
		Ubiquitin mediated proteolysis	3	4.3	6.00E-02
		Regulation of autophagy	2	2.9	9.90E-02
miR-762	534	Axon guidance	16	3	6.60E-07
		MAPK signaling pathway	16	3	2.90E-03
		Wnt signaling pathway	13	2.4	4.00E-04
miR-874	176	B and T cell receptor signaling pathway	4	2.3	2.20E-02
		MAPK signaling pathway	4	2.3	3.70E-01
mir-890	325	Wnt signaling pathway	7	2.1	6.50E-02
		ErbB signaling pathway	5	1.5	8.10E-02
miR-939	365	Calcium signaling pathway	10	2.4	1.30E-02
		ErbB signaling pathway	5	1.2	1.20E-01
		p53 signaling pathway	4	0.9	1.80E-01
		Wnt signaling pathway	6	1.4	2.20E-01

**Table 5:** **Main functions of downregulated miRNAs predicted by bioinformatic analysis**

miRNA	Putative target genes	KEGG pathway	Genes involved in the term	% of involved genes/total genes	***p***-value
(***Homo sapiens***)					
miR-1290	593	Pathways in cancer	17	2.9	4.00E-02
		Focal adhesion	14	2.4	7.90E-03
		Insulin signaling pathway	13	2.2	7.60E-04
		MAPK signaling pathway	12	2	1.90E-01
		ErbB signaling pathway	11	1.9	2.80E-04
miR-1915-3p	351	Wnt signaling pathway	8	2.3	5.60E-03
		Pathways in cancer	7	2	3.30E-01
miR-2861	170	Fc gamma R-mediated phagocytosis	4	2.2	3.60E-02
		MAPK signaling pathway	6	3.4	5.10E-02
		Arachidonic acid metabolism	3	1.7	6.80E-02
miR-3665	195	Neurotrophin signaling pathway	4	2.1	1.10E-01
		Insulin signaling pathway	4	2.1	1.30E-01
		MAPK signaling pathway	4	2.1	4.70E-01
miR-4257	197	-	-	-	-
miR-452-5p	327	Oocyte meiosis	8	2.3	1.30E-03
		Wnt signaling pathway	7	2	2.60E-02
		ECM-receptor interaction	5	1.4	3.80E-02
miR-513a-5p	980	MAPK signaling pathway	25	2.6	1.00E-02
		Pathways in cancer	24	2.4	1.30E-01
		Regulation of actin cytoskeleton	20	2	2.50E-02
miR-572	6	-	-	-	-
miR-629-3p	445	Pathways in cancer	10	2.3	2.10E-01
miR-765	548	Cytokine-cytokine receptor interaction	11	2	2.00E-01
miR-875-5p	181	MAPK signaling pathway	6	3.3	7.30E-02
		Spliceosome	5	2.8	2.10E-02
miR-940	-	-	-	-	-

## Discussion

The identification of novel anti-oxidant chemo-reagents is required to effectively treat or prevent alopecia due to androgen dysregulation or oxidative stress. Here, we used cell-based approaches, to demonstrate the potent anti-oxidant activity of the lignin derivative arctiin. Interestingly, pretreatment with arctiin drastically inhibited H_2_O_2_-induced decreases in viability in HHDPCs. Flow cytometry demonstrated that arctiin pretreatment also increased HHDPC resistance against cell death and G2 cell cycle arrest mediated by H_2_O_2_. Furthermore, those effects of arctiin were related to its anti-oxidative effect. Upregulation of intracellular ROS levels, which was mediated by H_2_O_2_, was dramatically inhibited following arctiin pretreatment. Recent reports have demonstrated that the loss of proliferative capacity in balding dermal papilla cells is associated with expression changes SA-β-gal and markers of oxidative stress [7]. We also found that arctiin inhibited H_2_O_2_-mediated upregulation of SA-β-gal activity in HHDPCs. These findings suggest that arctiin is a novel potent therapeutic agent for oxidative stress-induced cell dysfunction in HHDPCs.

Using miRNA microarrays, we identified 30 miRNAs that may have important roles in the arctiin-mediated protective effect against H_2_O_2_ in HHDPCs. Although further experiments are needed to validate miRNA expression levels to confirm the microarray data, we focused on the biological meaning of the altered miRNAs in our study because the cellular functions of miRNA are dependent on their target mRNAs. Therefore, we used several bioinformatic tools to understand the biological meaning of the altered miRNAs. Our bioinformatical analysis showed that the miRNAs altered in response to arctiin pretreatment before H_2_O_2_ stimulation are commonly involved in MAPK and Wnt signaling pathways. TAK1/MAP3K7 is a member of the MAP3K family, and it has been reported that impaired hair follicle morphogenesis and hair loss are mediated by TAK1/MAP3K7 deletion in mice [33]. Also, TAK1/MAP3K7 deficiency upregulates ROS levels, resulting in skin keratinocyte cell death [34]. Extracellular signal-regulated kinase (ERK), a member of the MAPK family, plays an important role in HHDPC proliferation. ERK signaling is activated by minoxidil, which is a widely used drug for treating androgenetic alopecia, and ERK inhibition blocks the anti-alopecia effect of the minoxidil [35]. In addition, our bioinformatic results showed that MAPK signaling was the most commonly targeted pathway for the downregulated miRNAs mediated by arctiin in HHDPCs (Table 5). This result indicates that MAPK pathway activation is important for HHDPC proliferation. However, our bioinformatic analysis revealed that the MAPK signaling pathway is also targeted by the upregulated miRNAs (Table 4), indicating that inhibition of MAPK signaling pathway might be involved in protective effects against ROS in HHDPCs. It has been reported that ROS activates ERK/MAPK, and ROS-mediated ERK activation induces apoptosis and senescence in several cell lines [36]. Although the molecular relationship between alopecia and MAPK signaling pathway remains to be investigated, our results indicate the possibility that regulating MAPK signaling might be important for treating or preventing alopecia.

Our bioinformatic analysis also revealed that WNT signaling pathway is putatively targeted by the miRNAs altered following arctiin treatment. Wnt signaling has also been implicated in alopecia. A recent study demonstrated that Wnt10A, which is a member of the Wnt family, is involved in the etiology of androgenetic alopecia [37]. A Wnt10A deficiency causes deregulation of the hair cycle by shortening the anagen phase, which is observed in androgenetic alopecia hair follicles [37]. Funato *et al.* demonstrated that H_2_O_2_-induced ROS can regulate Wnt/β-catenin signaling pathways [38]. Also, it was recently reported that the minoxidil-mediated anagen prolongation effect is due to β-catenin pathway activation [39]. Although further investigations are necessary to clarify the molecular interplay between ROS and Wnt signaling pathway in hair follicles and in patients with alopecia, our results suggest that arctiin-mediated anti-oxidative effects in HHDPCs may be involved in regulating Wnt signaling.

## Conclusions

In summary, our results demonstrate that arctiin regulates H_2_O_2_-induced cell death, cell cycle arrest, and ROS production in HHDPCs. Arctiin also inhibits H_2_O_2_-induced cell senescence. We identified 30 miRNAs that were significantly expressed following arctiin treatment, indicating that they may be involved in arctiin-mediated anti-oxidative processes. Taken together, our results provide evidence that the novel putative chemoreagent arctiin can prevent HHDP cell damage mediated by oxidative stress.

## Methods

### Cell culture and reagents

HHDPCs provided by Innoprot (Bizkaia, Spain) were purchased and maintained in Dulbecco’s modified Eagle’s medium (DMEM) containing 10% fetal bovine serum (HyClone; Thermo Fisher Scientific Inc., Waltham, MA, USA) and 1% penicillin-streptomycin (Gibco; Life Technologies, Grand Island, NY, USA) at 37°C and 5% CO_2_. Arctiin, propidium iodide (PI) for cell cycle analysis and 2′7′-dichlorofluorescein diacetate (DCF-DA) for intracellular ROS analysis were purchased from Sigma-Aldrich (St. Louis, MO, USA).

### Water-soluble tetrazolium salt (WST-1) assay

To analyze cell viability, HHDPCs were plated on 96-well culture dishes. After overnight growth, the cells were treated with various concentrations of arctiin (0–60 μM) for 24 h. WST-1 assay solution (EZ-Cytox Cell Viability Assay Kit, Itsbio, Seoul, Korea) was added for 40 min after the 24-h incubation. Cell viability was measured using an iMark microplate reader (Bio-Rad, Hercules, CA, USA) at 490 nm with a reference filter of 620 nm. The results are presented as mean percentage ± standard deviation (S.D.) of three independent experiments.

### PI-based cell cycle analysis

To analyze cells in different phases of the cell cycle, treated HHDPCs (4 × 10^3^) were gathered by trypsinization and fixed by adding cold 70% ethanol at −20°C for 1 h. After fixation, cells were stained by incubating with PI staining solution (50 μg/ml PI, 0.5% Triton X-100, and 100 μg/ml RNase) at 37°C for 1 h. The distribution of each cell cycle phase was determined by evaluating the intensity of fluorescence PI staining using the FL2-H channel of a FACSCalibur (BD Biosciences, Franklin Lakes, NJ, USA).

### DCF-DA-based ROS analysis

To analyze intracellular ROS levels in HHDPCs, treated cells were washed, trypsinized, and collected. Cells were diluted in 20 μM DCF-DA/phosphate-buffered saline (PBS) and incubated at room temperature for 1 h in the dark. After incubation, cells were washed once with PBS and subjected to flow cytometer-based fluorescence analysis using a BD FACSCalibur flow cytometer (BD Biosciences).

### β-galactosidase (β-Gal)-based cellular senescence analysis

To analyze the level of cellular senescence in HHDPCs after arctiin and H_2_O_2_ treatment, treated cells were gathered and fixed by the addition of 2% formaldehyde/0.2% glutaraldehyde. After fixation, senescence-associated β-galactosidase (SA-β-Gal) staining solution (Biovision, Milpitas, CA, USA) was added to the fixed cells and incubated at 37°C overnight. Senescent cells (positive blue color) were observed and counted using a bright-field microscope at × 200 magnification, and the percentages were determined.

### Microarray-based miRNA expression analysis

To investigate which miRNAs are altered in our study, treated HHDPCs were gathered and lysed using TRIzol reagent (Life Technologies) for total RNA purification. Total RNA was extracted from the lysed cells according to the manufacturer’s protocol and estimated its integrity and purity was estimated using an Agilent 2100 Bioanalyzer® (Agilent Technologies, Santa Clara, CA, USA) and a MaestroNano® microvolume spectrophotometer (Maestrogen, Las Vegas, NV, USA). We confirmed that the RNA samples had values integrity values higher than 8.0 and A260/280 and A260/230 values greater than 1.8. The qualified RNA samples were subjected to miRNA microarray analysis as described previously [40]. Briefly, RNAs were dephosphorylated and labeled with cyanine 3-pCp (Agilent Technologies). The labeled samples were dried and treated with GE Blocking Agent (Agilent Technologies) to reduce background or nonspecific binding to the probe onto the microarray. Then, the samples were hybridized to the SurePrint G3 Human v16 miRNA 8x60K microarray (Agilent Technologies) in the Agilent Microarray Hybridization Chamber (Agilent Technologies) for 20 h. After hybridization, the array was scanned using an Agilent SureScan Microarray Scanner (Agilent Technologies) and quantitated using Agilent Feature Extraction Software (version 10.7, Agilent Technologies). Derived data were analyzed using GeneSpring GX software, version 11.5 (Agilent Technologies). The data were filtered using flag-present and t-tests to identify miRNAs for further analysis. miRNA expression was evaluated by assessing the fluorescence ratio between two samples. Those displaying >2.0-fold increases or decreases were selected for further bioinformatic analysis.

### Bioinformatical tool-based biological analysis of miRNAs

To investigate the biological meaning of miRNAs with significantly altered expression, we used three kinds of bioinformatical tools: MicroCosm Targets Version 5 (http://www.ebi.ac.uk/enright-srv/microcosm/htdocs/targets/v5/), AmiGo 2 GO analysis tool and DAVID (Database for Annotation, Visualization and Interrogate Discovery, http://david.abcc.ncifcrf.gov/home.jsp) bioinformatics resources v6.7 [41]. Using the first tool, we predicted the putative target genes of the altered miRNAs, and the second tool was used to group target genes into four categories: aging, skin development, apoptosis, and cell proliferation. Finally, the targets were analyzed using the Kyoto Encyclopedia of Genes and Genomes (KEGG) pathway-based enrichment analysis program in the bioinformatic database DAVID. The Ensembl transcript ID lists of target genes were gathered and subjected to pathway analysis using the KEGG program in DAVID bioinformatic resources. The Ease score, which is a modified Fisher’s extract P-value, was fixed at 0.5 and meaningful KEGG pathways showing a value of >0.9% (percentage of involved target genes/total target genes) were selected.
